# Hematology thin smears perform equally to parasitology thick and thin blood smears for the diagnosis of *Plasmodium* and *Babesia* infections in a low prevalence setting

**DOI:** 10.1128/jcm.01601-24

**Published:** 2025-03-25

**Authors:** Janmesh Patel, Jill Schuett, Derrick J. Chen

**Affiliations:** 1 Department of Pathology, University of Wisconsin School of Medicine and Public Health 5232, Madison, Wisconsin, USA; 2 Clinical Microbiology Laboratory, UW Health, University Hospital 529799, Madison, Wisconsin, USA; 3 Department of Pathology and Laboratory Medicine, School of Medicine and Public Health, University of Wisconsin-Madison 189586, Madison, Wisconsin, USA; Mayo Clinic Minnesota, Rochester, Minnesota, USA

**Keywords:** malaria, *Babesia*, peripheral blood smear, thick smear, thin smear, diagnostics, low prevalence, parasitology, *Plasmodium*

## Abstract

Malaria and babesiosis are significant parasitic infections, requiring timely diagnosis to avoid severe complications. This study retrospectively compared hematology thin smears (HS) with parasitology thick and thin blood smears (PS), the gold standard for diagnosis, to evaluate HS’s performance in detecting *Plasmodium* and *Babesia* infections. Of 529 cases with paired HS and PS testing, HS demonstrated 93.3% sensitivity and 99.8% specificity, with 97.7% positive and 99.4% negative predictive values. When only considering new diagnoses, HS and PS were 100% concordant. No significant difference was found in percent parasitemia between HS and PS, highlighting HS as a reliable diagnostic tool in settings where PS or other diagnostic modalities may not be readily available.

**IMPORTANCE:**

This study demonstrates that hematology thin smears—often available in laboratories that may not have other means of diagnosing blood parasite infections such as parasitology thick and thin smears, rapid diagnostics tests, or polymerase chain reaction—are an accurate and reliable way to diagnose *Plasmodium* and *Babesia* infections in a low prevalence setting.

## INTRODUCTION

There are an average of 1,773 cases of malaria (*Plasmodium* species) and 1,762 cases of babesiosis (*Babesia* species) in the United States annually (1, 2). While timely treatment can lead to quick symptom relief for both diseases, severe complications can arise if therapy is delayed, including cerebral malaria, severe malarial anemia, coma, or even death for malaria, and thrombocytopenia, renal failure, and acute respiratory distress syndrome for babesiosis, particularly for those who are immunocompromised or lack a spleen (3–5). Given the risks associated with delayed treatment, accurate and timely diagnostic tests are crucial for early detection and appropriate management of both diseases.

Although the gold standard for the diagnosis of *Plasmodium* and *Babesia* infections is a thick and thin blood smear performed by a technologist trained in parasitology, this method has limitations (6, 7). Calculating parasite levels in blood smears manually can be labor-intensive and requires skilled and trained personnel (7). This technique is complex and can lead to diagnostic errors due to its demanding nature and potential limitations in the quality of microscopy and staining materials (8).

This study directly compared results from thin smears performed in a hematology laboratory (hematology smear or HS) to those obtained from thick and thin smears performed in a parasitology laboratory (parasitology smear or PS) to determine the performance of HS as an alternative approach for rapid diagnosis.

## MATERIALS AND METHODS

### Case selection and chart review

A retrospective chart review was conducted on all patients who had a paired in-house HS performed at UW Health and a PS performed at the Wisconsin State Laboratory of Hygiene (WSLH) from Jan 2018 to Oct 2023. Only cases where both HS and PS were performed on the same specimen were included for this study. For all cases included in the study, the details regarding whether treatment was administered, the type of treatment, and the timing of its administration were determined.

### Slide preparation

Four thin smears were prepared from peripheral whole blood collected in an EDTA tube within 1 hour of collection for a parasite screen. Smears were created using a HemaPrep Automated Blood Smearing Instrument, with each smear measuring 1–1.5 inches long with a feathered edge. Two thick smears were made by placing one drop of EDTA blood with a transfer pipette and spreading in a circular pattern until the blood spread to about the size of a penny in the center of each slide. A thick smear of proper density was one which, if placed wet over newsprint, the words could be barely read. The smears were allowed to completely air-dry before staining. Two thin smears were used for HS, and the remaining two thin smears and two thick smears were left unstained and sent for confirmatory testing at WSLH.

### Hematology thin smears

Two thin smears were stained using Colorwright Wright-Giemsa Stain and Sysmex SAS-100 Buffer (pH 6.8) on a Sysmex SP-10 automated stainer. The stain quality was assessed to ensure it clearly distinguished purple nuclear material and pink-red blood cells (RBCs) and was free of precipitate. If the stain quality was unacceptable, a new smear was prepared and stained. Immediately after the slides were prepared, two general technologists, trained to recognize *Plasmodium* and *Babesia* organisms but not to differentiate among genera or species, independently examined the two smears using a brightfield microscope using ×10, ×20, and ×50 objective magnifications to screen for large parasites and at ×100 magnification to screen for intraerythrocytic parasites. At ×100 magnification, 300 oil immersion fields were examined on each of the two thin smears. If no parasites were seen, the result was reported as “No blood parasites seen.” If parasites were detected, the result was reported as “*Plasmodium* or *Babesia* present” along with the percentage of parasitemia, rounded to the nearest whole number. HS smears were read and resulted during all hours of the day and week.

### Percentage of parasitemia for hematology thin smears

**Fig 1:**
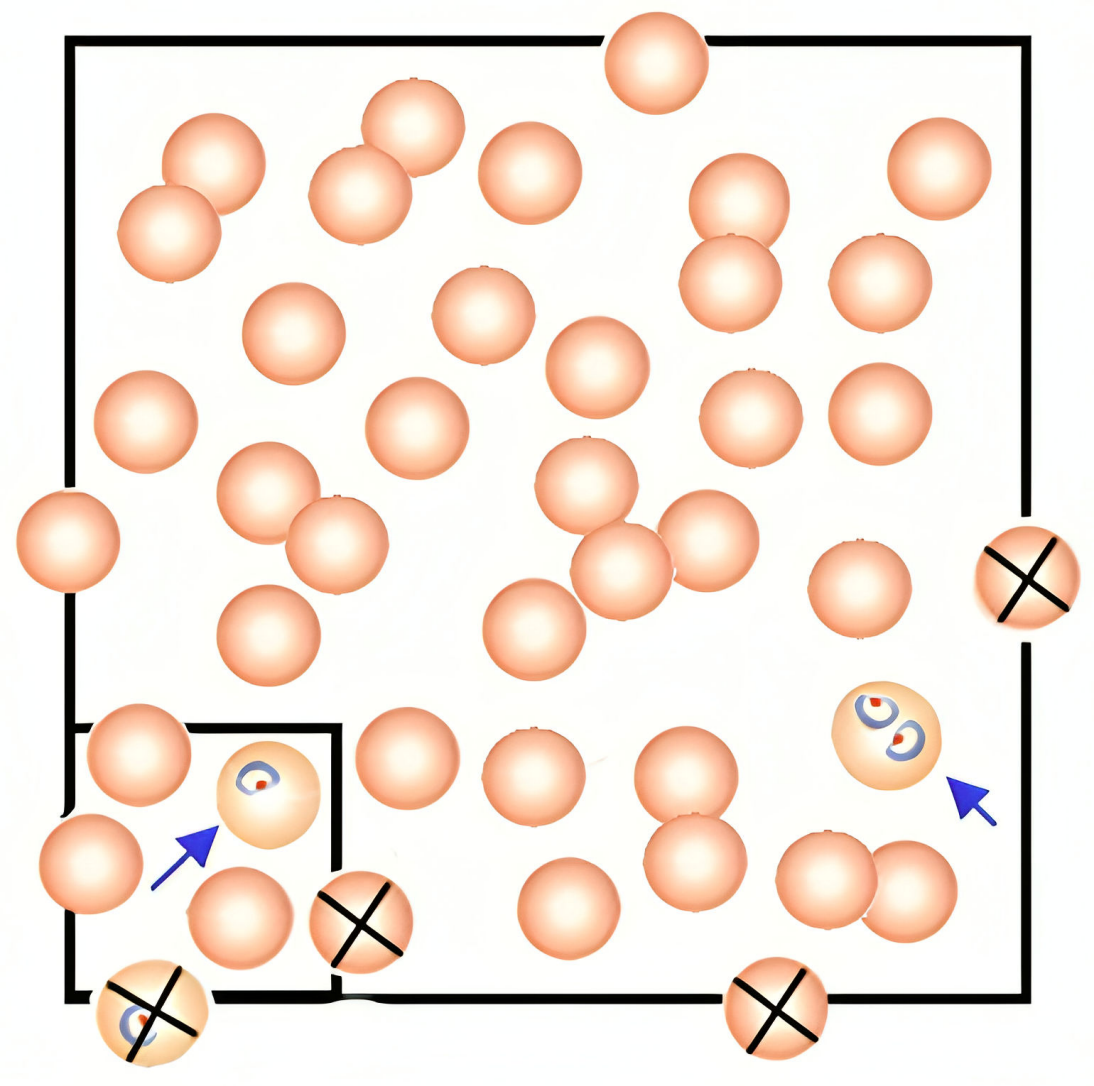
The percentage of parasitemia was determined by counting parasitized red blood cells (RBCs) in 1,000 RBCs using a Nikon Miller disc reticle under ×100 objective magnification.

The percentage of parasitemia was determined using a Nikon brightfield microscope. One ocular lens was replaced with a Nikon Miller disc reticle, which consisted of two squares: a small square and a large square, the latter being nine times the area of the former. This ratio had to be confirmed with the Miller disc reticle supplier since it was used in subsequent calculations. Using the Miller disc reticle, the number of parasitized RBCs was counted in 1,000 RBCs under ×100 magnification. Fields where just under half of the RBCs were touching were chosen. In each field, all the RBCs in the small square were counted, followed by the parasitized RBCs in both the small and large squares. Multiple parasites within a single RBC were counted as one. RBCs that overlapped the square line on the top and left were counted, while those that touched the square line on the right and bottom were excluded (Fig. 1). This process was repeated in different fields until the number of RBCs in the small square totaled at least 112, which corresponded to 1,000 RBCs in the larger square. The percentage of parasitemia was calculated using the following formula: (Number of parasitized RBCs in the large square × 100) ÷ (Total RBCs in the small square × 9). This process was repeated on the second smear. The parasitemia results from the two smears had to agree within two percentage points. The average of the two calculations was then rounded to the nearest whole number. If the parasitemia was less than 1%, it was reported as “<1%.” The additional smears, two thin and two thick smears, and blood were sent to WSLH for confirmation to verify the presence of parasites and further identify genus and species via microscopy and polymerase chain reaction (PCR) testing.

### Parasitology thick and thin blood smears

The two thin smear slides received at WSLH were fixed in absolute methyl alcohol by briefly dipping them in a Coplin jar, while the two thick smears were left unfixed. All thick and thin smear slides were air-dried and stained with 2.5% Giemsa stain by placing them in a Coplin jar for 45 minutes. Thin smears were washed by dipping slides 1–2 times into a Coplin jar with buffered water, while thick smears were placed into buffered water for 3–5 minutes. Both were air-dried and then read microscopically by a technologist trained specifically in parasitology. Slides were read at ×10, ×20, and ×100 objective magnifications to search for large and small parasites. Thin smear slides were carefully examined at ×100 magnification for at least 300 fields. Percent parasitemia was determined by counting infected RBCs and dividing by the average number of RBCs per field and the total number of fields read, and the calculated percent parasitemia was reported to the 1,000th of a percent. Laboratory-developed PCRs were used to confirm and identify organisms to the species level. Both species-level identification and percent parasitemia were included in the WSLH results reported. Findings from the PS thin smear and the PS thick smear were not differentiated from each other in the WSLH reports, so for the purpose of comparing HS to PS results, both PS thick and PS thin smear results from the same specimen were treated as one. PS smears were only read and resulted on weekdays during business hours and required specimen transport to WSLH, which also only occurred on weekdays.

## RESULTS

**Fig 2:**
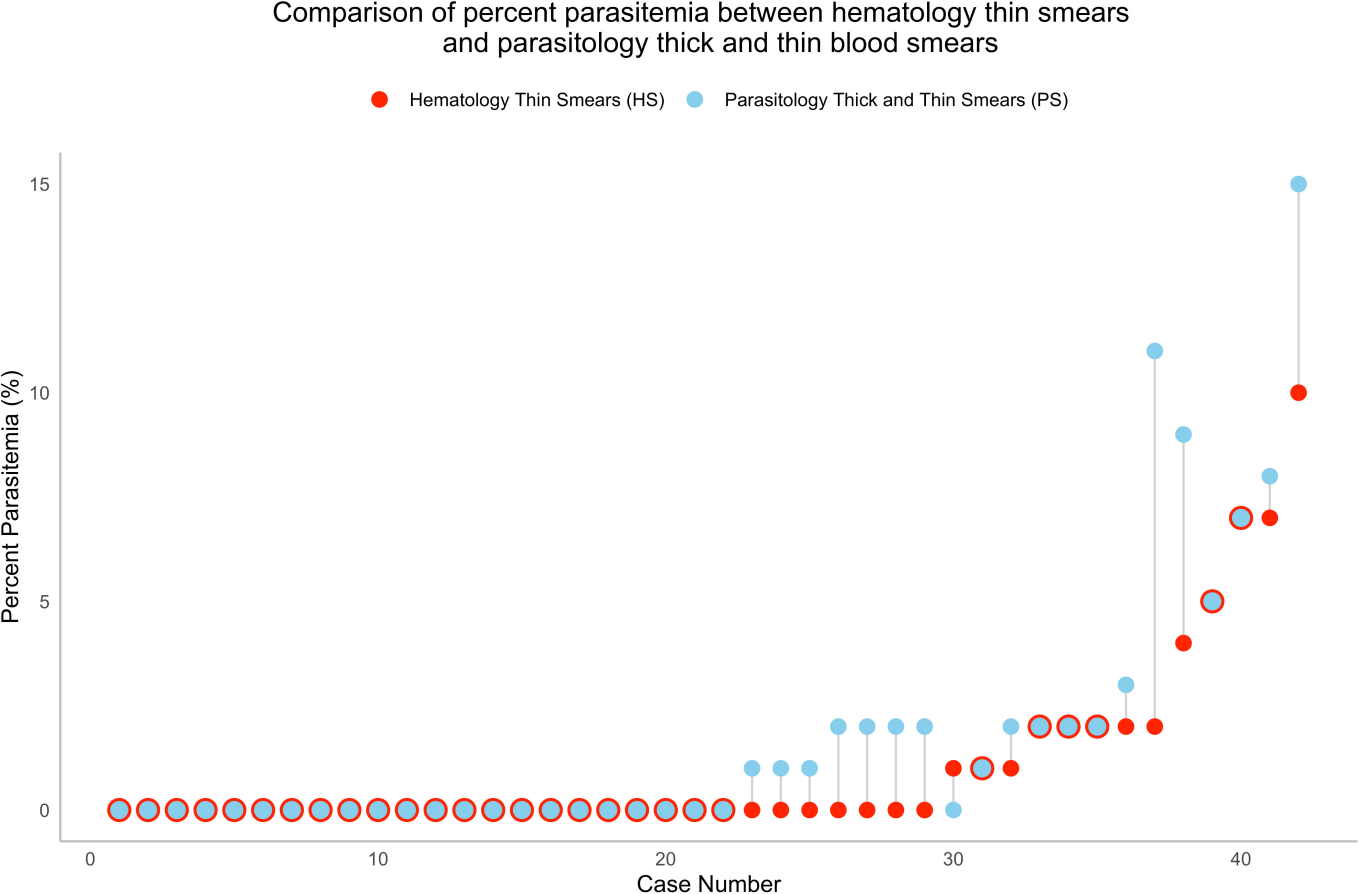
Scatter plot of the 42 cases in which the percent parasitemia values were reported for both PS and HS. For comparison, percents for PS were rounded to the nearest whole number in this figure to match HS, which were routinely reported as whole numbers. Difference between PS and HS was not statistically significant (*P* = 0.228).

There were 335 unique patients who were analyzed for this study. From these patients, a combination of initial diagnoses (335) and follow-up tests (194) yielded 529 instances during the study period where both PS and HS were performed on the same blood specimen. Four hundred eighty-three (91%) cases were PS−/HS−, and 42 (8%) were PS+/HS+, 3 (1%) were PS+/HS−, and 1 (<1%) was PS−/HS+. For all four discordant cases (3 PS+/HS− and 1 PS−/HS+), testing was performed as follow-up monitoring after a known diagnosis, and percent parasitemia values were all below the level of quantification. Using PS as the reference standard, HS sensitivity was 93.3% (42/45), specificity 99.8% (483/484), positive predictive value 97.7% (42/43), and negative predictive value 99.4% (483/486). For the 42 PS+/HS+ cases, there were 21 *Plasmodium* and 21 *Babesia* identified. Of those, 1st quartile, median (mean), and 3rd quartile percent parasitemia reported by PS were 0.02%, 0.1% (mean 1.2%), and 1.1% and by HS were <1%, <1% (mean 1.9%), 2%, respectively. There was no significant difference between PS and HS percent parasitemia values, and paired percent parasitemia results are shown in Fig. 2 (*P* = 0.2). The two HS slides read for each case all agreed within two percentage points of each other, so further arbitration was not needed. All patients with PS+ or HS+ received treatment. For initial diagnoses, treatment was administered after the HS was resulted, but prior to receiving the PS evaluation, demonstrating the value of the faster HS turnaround time.

## DISCUSSION

Overall, HS performed similarly to PS with high positive and negative predictive values in a low prevalence setting. When only considering new diagnoses of *Plasmodium* and *Babesia* infection, HS and PS showed 100% agreement. Occurrences of false-negative results for both PS and HS were rare and were only encountered in specimens from previously diagnosed patients already on therapy who were getting follow-up testing and had very low percent parasitemia levels. Reported percent parasitemia values for positive cases were comparable between the two methods and not significantly different.

The findings of this study have significant implications for clinical practice, particularly in settings where adequately trained personnel competent to interpret PS smears are not readily available (9). Moreover, proper training and supervision of technologists are essential for adequate sensitivity, as lack thereof can reduce accuracy (9, 10). The high sensitivity and specificity of HS suggest that it can be effectively used as a diagnostic screening tool for detecting *Plasmodium* and *Babesia* infections in a low prevalence setting, expediting the diagnosis and treatment of parasitic infections while confirmatory testing is pending. This can greatly benefit smaller hospitals and clinics that already perform HS but lack the resources for reading PS or maintaining rapid diagnostic tests (RDT). While HS was only reported as “*Plasmodium* or *Babesia* present” and percent parasitemia rounded to the nearest whole number, providers were able to use clinical assessment and epidemiology to select empiric therapy, which was appropriate for this patient population given the overall low level of parasitemia. This approach may also reduce the burden on specialized laboratories, such as WSLH, which may have the expertise to read PS and perform polymerase chain reaction but may not be able to provide rapid turnaround of results.

The diagnosis of *Plasmodium* and *Babesia* species can involve various methods such as PS, HS, RDT, and PCR. Microscopic examination of thick and thin blood smears is a traditional approach that is still used worldwide. Thick smears are thought to be more sensitive due to the higher concentration of blood, which allows for greater detection of parasites, but they can be challenging to interpret. Thin smears aid in identifying the parasite species and quantifying the infection, but they are less sensitive. In general, PS tends to have a sensitivity of 85%–90% and specificity of 99.5%–100% depending on the training and expertise of the examiner (10–13). Previous evaluations of thin smears compared to thick smears have shown considerable variability, with reported sensitivities ranging from 54.8% to 96.2% and specificities between 92.6% and 100% (10, 12, 14–17). On top of this, our study demonstrated that HS performed in a hematology laboratory by general technologists could achieve a sensitivity of 93.3% and specificity of 99.8%, rivaling PS performance, but with faster turnaround time. HS are used not only to detect parasites but also to identify morphological abnormalities in blood cells and are an affordable and more readily available diagnostic tool (18).

RDTs offer the advantage of quick results, which is important for patient management, and they do not require personnel trained in microscopy, making them particularly useful in resource-limited settings; however, they may lack sensitivity compared to microscopy and are not always able to determine parasite species or density (19). Although RDTs have slightly lower sensitivity and specificity, at 97% and 96% respectively, they remain essential in regions where resources and trained personnel for interpreting PS and HS are unavailable (11, 20, 21). Additionally, these figures may vary depending on the RDT manufacturer and the specific *Plasmodium* species being tested; for instance, our study found HS sensitivity (93.3%) to be within the range of reported sensitivities for BinaxNow RDT (92.9%–99%; the manufacturer claims an average sensitivity of 97.7% among *Plasmodium* species) (19, 22, 23). The sensitivity and specificity for *Plasmodium falciparum* are most consistent with the previously stated values, but for *Plasmodium vivax* and *Plasmodium ovale*, RDT sensitivity can drop to as low as 66%–88% and 86%, respectively (21). Thus, in most cases, either PS or HS is preferable. PCR offers nearly 100% sensitivity and specificity, making it the most accurate method for detecting parasitic infections, especially for confirming species and detecting drug resistance (20, 24–26). It can identify parasitemia as low as 2–5 parasites/μL, compared to the 100 parasites/μL detected by RDT and 50–500 parasites/μL by HS and PS (20, 27). However, due to the complexity of the required equipment, reagent maintenance, and the need for highly qualified personnel, PCR is not readily available, and specimens often must be sent out to specialized laboratories, unlike RDT and HS, which are more accessible in clinical settings (20).

This study demonstrated that HS performed comparably to PS in diagnosing *Plasmodium* and *Babesia* infections, making HS a viable alternative in low prevalence settings. HS’s high sensitivity and specificity, along with its strong agreement with PS results, suggest that it can serve as an efficient and reliable diagnostic tool, especially in settings where PS, RDTs, and PCR are not available. This study’s findings are limited by the relatively small number of positive cases (*n* = 46), and further investigation with larger sample sizes is needed to validate these results.
